# Contribution of *Amaranthus cruentus* and *Solanum macrocarpon* Leaves Flour to Nutrient Intake and Effect on Nutritional Status of Rural School Children in Volta Region, Ghana

**DOI:** 10.1155/2020/1015280

**Published:** 2020-06-02

**Authors:** Godfred Egbi, Mary Glover-Amengor, Margaret M. Tohouenou, Francis Zotor

**Affiliations:** ^1^Nutrition Department, Noguchi Memorial Institute for Medical Research, College of Health Sciences, University of Ghana, Legon, Accra, Ghana; ^2^Council for Scientific and Industrial Research-Food Research Institute (CSIR-FRI), Accra, Ghana; ^3^Department of Nutrition and Food Science, University of Ghana, P.O. Box LG 134, Legon, Accra 12, Ghana; ^4^School of Public Health, University of Health and Allied Sciences, PMB 31, Ho, Ghana

## Abstract

**Background:**

Plant-based foods are staple diets and main micronutrient sources of most rural Ghanaian households. The objective of this study was to determine the effect of *Amaranthus cruentus* and *Solanum macrocarpon* leafy vegetable flour on micronutrient intake and nutritional status of rural Ghanaian school children.

**Method:**

This study was a randomized controlled trial that consisted of baseline data collection and a three-month nutrition intervention feeding program. Two groups of 53 children, age 4–9 years, involved in the Ghana School Feeding Program took part in the study. An experimental group consumed *Amaranthus cruentus* and *Solanum macrocarpon* leaves flour (ACSMLVF) stews and soup. The control group consumed stews and soup without ACSMLVF. Haemoglobin and serum vitamin A concentrations were determined. Dietary and anthropometric data were collected and analysed. Participants were screened for malaria parasitaemia and hookworm.

**Results:**

Anaemia was present in 41.5% and 37.3%, respectively, of the intervention and control groups at baseline. It was present in 28.3% and 53.3%, respectively, at the end of the study. This was significantly different (*p*=0.024). There was a low vitamin A concentration in 66.0% and 64.7% at baseline and 20.8% and 23.4% at the end of the study in the intervention and control groups, respectively. The mean iron, zinc, and provitamin A (beta-carotene) intakes of the intervention group were 14.2 ± 7.1 mg, 5.7 ± 2.1 mg, and 214.5 ± 22.6 *μ*g, respectively, at baseline. Those of the control were 13.7 ± 6.1 mg, 5.4 ± 2.1 mg, and 210.6 ± 20.1 *μ*g, respectively. At the end of the study, the mean intake of iron, zinc, and beta-carotene for the intervention group was 24.1 ± 10.9 mg, 13.8 ± 8.2 mg, and 694.2 ± 33.1 *μ*g, respectively. The intake of these micronutrients for the control at the end of the study was 14.8 ± 6.2 mg, 5.9 ± 2.3 mg, and 418.4 ± 34.7 *μ*g, respectively.

**Conclusion:**

Consumption of ACSMLVF stews and soup increased iron, zinc, and beta-carotene intakes. Anaemia prevalence was lower in the intervention group at the end of the study.

## 1. Introduction

Micronutrient deficiencies are a major public health problem amongst vulnerable groups such as infants, children, pregnant, and lactating women in the developing world [1]. For good health, a balanced diet consisting of starchy foods as well as protein and micronutrient rich foods is essential [2], since such a diet has negative association with the risk of chronic diseases [3].

Vegetables and fruits are rich sources of minerals, vitamins and phytonutrients in sub-Saharan Africa [4, 5]. Plant-based foods are staple diets and the main source of micronutrients for most rural Ghanaian households. Vegetables and fruits are abundant and largely consumed during the rainy season. The availability, accessibility, affordability, and consumption of vegetables become a challenge during the dry season in poor households in Ghana and other West African countries. Consequently, poor household members are unlikely to meet their Recommended Dietary Allowances [6] for micronutrients in the dry season.

Due to high water activity, green leafy vegetables are perishable. Heat-processing methods (sun or solar and mechanical drying) reduce their moisture contents which make it feasible to process them into flour, so they can be preserved for use in the dry season. Analysis made on the vitamin composition of the dry leaves of *Amaranthus cruentus (*known locally as Aleefu) and *Solanum macrocarpon* (known locally as Gboma) indicates appreciable levels of beta-carotene (63.0 mg/100 g and 64.0 mg/100 g) and ascorbic acid (1.5 mg/100 g and 2.4 mg/100 g), respectively [7, 8]. In Ghana, the leaves of these plants are used in soup and stew preparations just as spinach is used in other parts of the world. This study sought to determine the contribution that consumption of stews and soups made from *Amaranthus cruentus* and *Solanum macrocarpon* leaves flour will make to total nutrient intake and the effect on nutritional status of rural Ghanaian school children.

## 2. Materials and Methods

### 2.1. Study Design

The study was a pretest and posttest design. It consisted of baseline data collection and a three-month nutrition intervention feeding program. The study consisted of an intervention and a control group. The children (of a community basic school complex) were randomized by simple random sampling into the two groups. The intervention group received school lunch stews and soups prepared with *Amaranthus cruentus* and *Solanum macrocarpon* flour (ACSMVLF). The control group had no ACSMVLF in their stews and soups. The feeding was done on every weekday and lasted for a period of three months.

### 2.2. Study Area

The study was carried out in the Adaklu District of the Volta Region. The capital town is Adaklu-Waya [9]. The district shares boundary at the west with Ho Municipality, south with Agotime-Ziope District, north with Akatsi District, and east with Ketu North District. The district has an area of 709 km square [9]. A population and housing census carried out in 2010 [9] showed that the population of Adaklu District was 36,391 (49.0% male) [9]. The majority of the people (78%) engaged in peasant farming. The food staples grown were maize and cassava. Other crops included cowpea, groundnut, tomatoes, garden eggs, pepper, and okra. To a small extent at the household levels were sheep, goat, and poultry rearing.

### 2.3. Study Population

The study population consisted of pupils 4–9 years of age attending the Adaklu-Kodzobi basic school. The two groups were similar in age, physiological makeup, and the Recommended Dietary Allowances (RDIs) for micronutrients [6]. At the time of the study, the subjects were also participating in the Ghana School Feeding Program (GSFP) where school lunch is provided.

### 2.4. Sample Selection Criteria

A child qualified to enroll in the study if he or she was aged four to nine years; attending school regularly; if parents gave written consent and their children provided assent to participate; enrolled in the Ghana Government School Feeding Program; and had no history of allergy to consumption of vegetables or vegetable flours as self-reported (or guardian).

### 2.5. Sample Size Determination and Sampling

This was a pilot study based on a sample size of 90 participants estimated with 0.6 g/dl mean change in haemoglobin concentration with a standard deviation of 1.2 (unpublished) and assumed a standardized effect size of 0.4 and a power of 80% with a significance level of 0.05. Assuming an expected drop-out rate of 18%, the sample size was increased, to give 53 participants per group. A sample frame was constructed of all the children who met the study criteria. The children were randomized by simple random sampling.

### 2.6. Chemical Analysis

The various stews and soups were analysed for their moisture, ash, protein, fat, iron, and zinc contents using standard protocols [10]. Beta-carotene content of the stews and soups was determined by the HPLC procedure of Rodriguez-Amaya and Kimura [11]. Triplicate determinations were made using the means as true values.

### 2.7. Vegetable Flour Preparation and Feeding of Participants

Fresh *Solanum macrocarpon* and *Amaranthus cruentus* leaves were purchased from urban market gardeners in Accra. The fresh leaves were soaked in 1% sodium chloride solution, rinsed with running tap water, and later dried in a mechanical air oven at 45°C for 10 hours. The dried leaves were ground separately into fine flour using a blender (Philips HR 2113, Netherlands). Both flours were mixed into a uniform blend of composite flour containing equal proportions (1 : 1 wt/wt) of each kind of vegetable with a cake mixer (Philips Viva Mixer HR 1565, Netherlands). Two hundred and thirty grams of *Amaranthus cruentus* and *Solanum macrocarpon* leafy vegetable flour (ACSMLVF) was packaged in an airtight plastic (polythene) bag and stored in dark cardboard. Bean stew, tomato stew, or groundnut soup was prepared separately with tomato paste (200 g), ground pepper (15 g), onion paste (35 g), smoked anchovies powder (100 g), and iodized salt (40 g) with or without the composite flour of the (ACSMLF). Groundnut oil (400 g) was used to prepare either the beans or tomato stew. Groundnut paste (400 g) was used to prepare groundnut soup. An amount of 230 g of ACSMLVF was added to the stew or soup for the intervention group. The food for the control group did not contain any ACSMLVF. Each participant in the intervention group was given 50 g of tomato and 100 g of bean stews and 95 g of groundnut soup fortified with ACSMLVF. The ACSMLVF-fortified tomato stew was served thrice a week at lunch break. The ACSMLVF-fortified bean stew was served once a week, and the ACSMLVF-fortified groundnut soup was also served once a week. Each participant in the control group was given the same quantity and frequency of the tomato and bean stews and groundnut soup without ACSMLVF. The stews and soup were eaten with 230 g of boiled plain rice (twice a week), Ga-kenkey (twice a week), and banku once in a week. Ga-kenkey and banku are fermented and cooked corn dough meals. To prevent trading of the served meals, participants from the intervention and the control groups were identified with green and yellow buttons, respectively, on their breast pockets. Each group was served with food at a different location in the school premises, and they were supervised by teachers and research assistants to maintain compliance. The study was carried out from mid-September–mid-December after the major rainy season.

### 2.8. Data and Biological Sample Collection and Analyses

Semistructured questionnaires were used to collect data on characteristics of the participants. Dietary data were captured through a food frequency questionnaire, 24-hour recall, and direct weighing of foods consumed. At every meal time, left over foods for individual participants were weighed and subtracted from the amount of meal served to account for actual food intake of each participant.

Food measures such as cups, spoons, ladles, and balls were provided to assist the respondent in assessing portion sizes of the food consumed. Portion sizes of the foods consumed were then estimated and recorded. Prices of purchased foods were also recorded. To enhance the accuracy of estimation of the weight of purchased foods, samples of purchased foods were weighed with an ultramodern electronic scale (Soehnle Plateau 56377, LEIFHEIT AG Nassau, Germany) to obtain the weight of the foods. The total amounts of specific food consumed were computed manually. The amounts of the various nutrients: protein, fat, iron, zinc, vitamin C, folate, and vitamin B12 were calculated based on 100 grams portion sizes with the help of Microsoft Office Excel 2007 and the food composition table [12, 13]. The weights of individual participants were measured to the nearest 0.1 kg in triplicate with the Seca digital weighing scale (Seca scale 803), according to a WHO 2006 protocol [14]. The actual weight of a participant was the average of triplicate measurements to the nearest 0.1 kg. The height of each participant (in triplicate) to nearest 0.1 cm was taken with a stadiometer in a standing position in accordance with standard procedures [14]. The average of three readings was recorded as the true value. The weight and height measurements were done at baseline and the end of the study.

A phlebotomist from the Parasitology Department of the Noguchi Memorial Institute for Medical Research (NMIMR), University of Ghana, Legon, collected 2 ml of venous blood by venepuncture from every participant early in the morning before breakfast. Blood samples were collected into serum separating tubes coated with gel (a clot activator). Blood samples collected were transported on ice packs to the Nutrition Department's laboratory, NMIMR. At the laboratory, each blood sample was centrifuged at 2,000 rpm for 15 min. And duplicate serum aliquots were prepared into Eppendorf tubes and stored under −80°C until analysed. The venous blood samples collected were used immediately in the field to determine haemoglobin concentrations by a haemoglobinometer (Hb 201+) (HemoCue AB Angelholm, Sweden). The average of two readings was taken as the actual haemoglobin concentration of each participant. The serum sample from each participant was used to determine his or her serum vitamin A concentration by HPLC technique, according to the established protocol of NMIMR [15]. The standard Giemsa staining technique [16] was used to screen for malaria parasite infection in the participants in prepared blood film slides. Stool samples were collected and used to screen for presence of hookworm by the Kato–Katz technique [17].

### 2.9. Data Analysis

The amount of intake of nutrients was calculated using the Ghana Food Composition Table, Ring database, and West African FAO database. All measured variables were checked for normality. Haemoglobin, serum retinol, weight, and height values were normally distributed. Anaemia was defined as haemoglobin (Hb) concentration < 10.9 g/dl, mild—Hb 10.0–10.9 g/dl, moderate—Hb 7.0–9.9 g/dl, and severe—Hb < 7.0 g/dl for children below the age of five years [18]. For children 5–9 years of age, it was defined as mild anaemia—Hb 11.0–11.4 g/dl, moderate—Hb 8.0–10.9 g/dl, and severe—Hb < 8.0 g/dl–<80 [18]. Summary values were presented as means plus or minus standard deviations and percentages. Student's *t*-test was used to compare mean values of control and intervention groups for any significant difference. Within-group individual differences were determined between baseline and end of the study periods using the paired *t*-test. Binary logistic regression was used to establish association of anaemia with other factors. One-way analysis of variance (ANOVA) was used to compare the mean nutrient composition of the various stews and soups. Significance was set at *p* ≤ 0.05. The amount of nutrients consumed by the study participants was compared to the Recommended Daily Intakes (RDIs) of the various nutrients.

## 3. Ethical Approval

The Institutional Review Board (IRB) of the Noguchi Memorial Institute for Medical Research, College of Health Sciences, University of Ghana, Legon, gave ethical approval (NMIMR-IRB CPN001/14-15) for the study to be conducted. Participants and guardians gave written or thumb print consent to take part in the study.

## 4. Results

### 4.1. Background Characteristics

Fifty-three children were recruited for each group at baseline, but only 51 children recruited for the control group provided biological samples. Two children declined for religious reasons. The study participants had similar background characteristics (Table 1). There was no significant difference between the two groups in terms of gender, mean age, and possession of a backyard garden to provide vegetables for the households. Just under half (47.1%) were male. Almost all (99.0%) of the parents (or guardians) were in the 20–60-year age range. Most (94.2%) of the parents (guardians) were earning an income of ≤ GHȻ500 ($178.60) per month (Table 1). Only 4.8% of the parents (guardians) did not have formal education. The majority (68.3%) of the study participants' households held food taboos of cultural significance; for example, an aversion to eating snails and reptiles. The level of stunting (HAZ score < −2 standard deviation) in the intervention group was 13.2% and 17.6% in the control group at baseline (Table 1). Wasting was present in 11.3% of the participants in the intervention group (WAZ score < −2 standard deviation), and 15.7% of the control wasted. Thinness (BMIAZ score < −2 standard deviation) at baseline was seen in 5.7% and 3.9% in the intervention and control groups, respectively. The level of anaemia in the children at baseline was 39.4% (Table 1). It was 41.5% and 37.3% in the intervention group and the control, respectively. The level of moderate anaemia was 9.4% in the intervention and 3.9% in the control group. The overall presence of low vitamin A concentration (<20 *μ*g/dl) [19] was 65.4%; more specifically, it was mild—15–20 *μ*g/dl (26.9%), moderate—10–14.9 *μ*g/dl (21.2%), and severe—<10 *μ*g/dl (17.3%). The presence of malaria parasitaemia was 35.6% at baseline. No participant had hookworm infection. Almost every study participant (98.1% in the intervention group and 100.0% in the control group) reported eating three times every day (Table 1). Only 13.2% and 9.8% of participants in the intervention group and the control, respectively, had a high dietary diversity score, eating diets made from the six food groups (cereals; legumes, nuts, and oils; fruits and vegetables; roots and tubers; meat, poultry, and fish; and fats and oils) available in Ghana. Many of the participants, 71.7% and 76.5% from the intervention and the control groups, had medium dietary diversity score, eating meals made from four or five food groups.

The results indicate that study participants whose parents earned the minimum income (1.0–499.0 cedis per month, equivalent to 1.0–178.6 USD) were two times more likely to be anaemic (OR: 1.95; CI: 0.22–0.86; *p*=0.039) compared to those whose parents earned at least 1000 cedis a month (Table 2). The participants with low serum retinol concentrations were 1.7 times more likely to have anaemia than those who had normal serum retinol levels (OR: 1.68; CI: 0.10–0.99; *p*=0.049) (Table 2). Other factors included in the binary logistic model, parental education status, parental marital status, and nutritional, infection, and dietary intake status of participants, were associated with anaemia but not significant (Table 2).

### 4.2. Nutrient Intake

The results from weighed food records showed that stews and soup fed to the intervention group had a much higher content of protein, fat, ash, iron, and zinc compared to those of the control group (Table 3). There was no significant difference in protein intake of the study groups at baseline. The mean protein intakes of the intervention and control groups at the end of the study were 38.1 ± 9.1 g and 32.6 ± 7.5 g, respectively.

The mean iron, zinc, and provitamin A (beta-carotene) intakes of the intervention group were 14.2 ± 7.1 mg, 5.7 ± 2.1 mg, and 214.5 ± 22.6 *μ*g, respectively, at baseline. Those of the control were 13.7 13.7 ± 6.1 mg, 5.4 ± 2.1 mg, and 210.6 ± 20.1 *μ*g, respectively (Table 4). At the end of the study, the mean intake of iron, zinc, and beta-carotene for the intervention group were 24.1 ± 10.9 mg, 13.8 ± 8.2 mg, and 694.2 ± 33.1 *μ*g, respectively. The values for the control group were 14.8 ± 6.2 mg, 5.9 ± 2.3 mg, and 418.4 ± 34.7 *μ*g, respectively. The stews and soup made from *Amaranthus cruentus* and *Solanum macrocarpon* leaf flour (ACSMLVF) contributed on the average of 580.0 *μ*g of beta-carotene to each participant in the intervention group. The amount was estimated based on the amount of various stews and soups provided (Table 3). The control also derived an estimated average of 220.0 *μ*g of beta-carotene from their stews and soups. The percentage of participants in the intervention group who met their DRI for iron, zinc, and provitamin A were 83.0%, 89.4%, and 94.3%, respectively, at the end of the study. The values in the control group were 61.7%, 53.2% and 60.9%, respectively. The change in mean intakes of iron, zinc, and beta-carotene for the intervention group was 9.9 mg, 8.1 mg, and 479.7 *μ*g, respectively, and for the control group, 1.1 mg, 0.5 mg, and 207.8 *μ*g, respectively. There were no significant differences in the changes in mean intakes of vitamin C, folic acid, and vitamin B_12_ of study participants from baseline to the end of the study for both groups (*p* > 0.05). None of the children met their DRI for folic acid at baseline and at the end of the study (Table 4).

### 4.3. Nutritional and Infection Status of Study Participants

At the end of the study, 39.0% of the participants were still anaemic (Table 5). The level of anaemia was 28.3% in the intervention group and 53.3% in the control (*p*=0.024). The level of mild anaemia was 24.5% and 46.8% in the intervention and the control groups, respectively (*p*=0.022). The overall prevalence of low vitamin A concentration among the participants at the end of the study was 21.2% (Table 5). No subjects in the intervention group had a moderately or severely low vitamin A concentration in the vitamin A level at the end of the study. However, this was present in 4.3% and 2.1%, respectively, of subjects in the control group (Table 5). At the end of the study, no significant difference was observed in the prevalence of stunting, wasting, and thinness as well as overweight and obesity between the two groups (*p* > 0.05). There was no significant difference in the prevalence of malaria parasitaemia between the two groups at the end of the study. No hookworm infestation was observed in the participants.

### 4.4. Dietary Diversity Score of Study Participants

The dietary diversity score (based on baseline data and data collected during the intervention) was calculated from 24-hour dietary recall and food frequency data based on nine food groups [20]. The mean dietary diversity score was 4.3. The majority of the participants in both study groups had a medium dietary diversity score (consumed food from 4 to 5 food groups). Only 13.2% and 9.8% of the participants from the intervention and control groups, respectively, had a high diversity score (≥6 food groups). The dietary diversity score for children in the intervention group did not differ significantly from the control group (*p*=0.829). Findings from the dietary diversity tertile showed that the majority of the participants from both groups consumed starchy staples, other vegetables, dark green leafy vegetables, and fish. Fruits and dairy products were scarcely eaten by the study participants (Table 6).

## 5. Discussion

The study established various forms of malnutrition among the participants, including anaemia, low vitamin A, wasting, stunting, thinness, overweight, and obesity. There was a higher prevalence of low vitamin A concentration in the intervention group than in the control group, though it was not statistically significant. The high prevalence of low vitamin A at baseline may be attributed to inadequate intake of foods rich in vitamin A, malaria, and other infections or inflammations that the study did not investigate. Studies have shown that infections and inflammations affect retinol and carotenoid metabolism and biomarkers of vitamin A status [21, 22]. In view of those research findings, the prevalence of low levels of serum retinol (vitamin A deficiency) may be overestimated in tropical countries such as Ghana. The findings have shown that 20.8% of the participants in the intervention group who consumed *Amaranthus cruentus* and *Solanum macrocarpon* leaf flour (ACSMLVF) had prevalence of mildly but not moderately low vitamin A levels at the end of the study as had been observed in the controls. The level of decreased prevalence of low serum retinol observed in the control during the intervention may be attributed to likely decline in general infections other than malaria (that the present study did not investigate), and that might have been intense during the period preceding the intervention. The food frequency data show that both study groups had scarce access to some fruits, vegetables, fats, and vegetable oils eaten away from school probably during breakfast and dinner. Those might be the source of provitamin A foods available to both groups. The control diets (stews and soup) to some extent also provided some level of beta-carotene (averagely, 220 *μ*g) in addition to some amount (198 *μ*g) captured from 24-hour recall for the control group. It is the total intake of beta-carotene at the end of the study that leads to the significant difference observed within the controls. The intervention group had on average 114 *μ*g beta-carotene based on 24-hour recall and extra 580 *μ*g beta-carotene from the intervention diets (stews and soup). The significant difference between the two groups could be attributed to ACSMLVF.

The findings have also demonstrated that consumption of ACSMLVF results in a significant reduction in the prevalence of anaemia. The findings suggest that ACSMLVF may be a simple but innovative food that can be used for food fortification (or modification) in order to protect against anaemia in school children during school feeding programs when fresh green vegetables are out of season. A similar study carried out among school children using fresh carotene-rich vegetables was found to increase haemoglobin concentration and reduce the prevalence of anaemia [23]. ACSMLVF is also a good source of beta-carotene. It may have helped improve the haemoglobin concentration and increased the vitamin A stores of subjects in the intervention group [24]. Our findings lend support to the suggestion of Maramag et al. [23] that beta-carotene may have compartmentalized effects on iron metabolism by enhancing incorporation of iron into haemoglobin.

The causes of anaemia are multifactorial. We suggest, similarly to Idohou-Dossou et al. [25], that the residual anaemia in the intervention group could be due to deficiencies of other nutrients (such as folic acid and ascorbic acid) in addition to iron and vitamin A, antinutritional factors (polyphenols and phytic acid), and infection (malaria in the case of the present study). As ACSMLVF is a good source of micronutrients (iron, zinc, and beta-carotene), it may have the potential to control nutritional anaemia in relation to deficiencies of iron, zinc, and vitamin A. Malaria infection is known to promote anaemia. It is possible, as suggested by previous studies [25], that malaria and other infections could partially explain the prevailing anaemia condition of the intervention group at the end of the study. The prevalence of malaria infection increased (but nonsignificantly) in the participants at the end of the study. That could probably be due to noncompliance to use treated mosquito bed nets even though the investigators provided each participant with a bed net. Binka and Akweongo [26] have emphasized the effective use and the potential of long-lasting insecticide nets to kill or prevent mosquitoes from biting individuals. It was not the owning of such nets that were protective against malaria. Even though the association between malaria and anaemia was not statistically significant in this study, participants with this infection were 1.6 times more prone to anaemia than those without it. Another study [27] conducted among children in the same region as the current study in Ghana found that those with malaria infection had a higher risk of being anaemic than those without the infection. Undernutrition and overnutrition were prevalent among the participants at the start and end of the study, but without any statistical difference.

Undernutrition is a challenge to the study participants as it is with other Ghanaian children in poor settings [28–30]. Ghana is in West Africa, a region with little progress in the past two decades to reduce the prevalence of stunting in children [31]. Nevertheless, two countries in the region (Ghana and Liberia) have been making an effort to reduce the prevalence of underweight to half the prevailing regional figure [32]. Based on the results of the present study, ACSMLVF consumption is not an appropriate strategy for controlling malnutrition within three months. It is suggested that its effectiveness to minimize or control malnutrition needs to be investigated further beyond three months. This was outside the scope of this study.

The addition of ACSMLVF effectively improved the protein, iron, zinc, and beta-carotene content of the stews and soup provided for the intervention group. This led to a significant increase in the dietary intake of these nutrients by the intervention group compared to the controls. The study findings confirm that leafy vegetable flours [33] may be rich sources of micronutrients (iron, zinc, and beta-carotene) just like their fresh forms [5, 8, 34]. The regular consumption of ACSMLVF could allow an individual or a population to meet the Recommended Daily Intake (RDI) of these nutrients. The findings show that improvement in the iron, zinc, and beta-carotene content of the stews and soup by the addition of ACSMLF resulted in a significant increase in the number of participants in the intervention group who met their RDI for iron, zinc, and beta-carotene. An important area of concern to be considered in further research is the bioavailability of these nutrients; this was not investigated in this study. The bioavailability of iron and zinc would to some extent depend on the presence of antinutritional factors (polyphenols and phytic acid) in the ACSMLVF. There was no significant difference in the dietary intake of water-soluble vitamins (ascorbic acid and folic acid) within and between the study groups. It is possible that much of these water-soluble vitamins were lost during powder and food preparation. It is suggested that ACSMLVF consumption be supplemented with other rich sources of water-soluble vitamins in order to prevent their deficiencies.

The major sources of food for the study participants as indicated by the baseline results were starchy staples (cereals, roots, and tubers), legumes, and fish. Fruits, fats, oils, and dairy products were limited in their normal diets. A previous study [35] conducted among Ghanaian school children also established that fruits, fats, oils, and dairy products were scarcely consumed by school children. Most of the participants in both the intervention (71.7%) and the control groups (76.5%) had a medium dietary diversity score. The findings indicate that only small fraction of the participants (9.8–13.2%) consumed highly diversified diets. These children were able to consume diets made from the six food groups available in Ghana. The findings also indicate that diets of all participants were predominantly made of plant staples (cereal, root, tuber, and legumes). According to Zimmermann et al. [36], populations whose habitual diet is plant based may be at high risk of iron deficiency. The reason for this is that plant iron (nonhaeme iron) is poorly bioavailable for absorption and utilization. However, iron from meat or meat products (haeme iron) is readily bioavailable and absorbable. Antinutrients such as phytic acid and polyphenols are known to bind to the nonhaeme iron and inhibit its availability. The dietary data reveal that the participants consumed little meat, poultry, and poultry products.

Monthly income and serum retinol status are economic and nutrition factors that are independently and significantly associated with anaemia among the study participants. The parental monthly income might dictate the intake by the participants of foods rich in iron, vitamin A, beta-carotene, zinc, vitamin C, and folic acid. Rich food sources of these micronutrients are known to prevent nutritional anaemia [36]. A low serum retinol status was significantly associated with anaemia in the participants. Low serum retinol status is an indicator of vitamin A deficiency; participants with low serum retinol concentration were 1.7 times more likely to be anaemic compared to those with normal serum retinol. The findings give support to existing knowledge that the causes of anaemia are multifactorial [36–39]. The results of this study show that anaemia is associated with nutritional factors, infection, and socioeconomic factors which should be considered for further study.

## 6. Conclusions

The addition of *Amaranthus cruentus* and *Solanum macrocarpon* leafy vegetable flour (ACSMLVF) to school meal stews and soup improved the content and intake of iron, zinc, and beta-carotene of the study participants. The consumption of ACSMLVF allowed for at least 85% of the study participants to meet their Recommended Daily Intake of iron, zinc, and beta-carotene. Consumption of ACSMLVF-fortified stews and soup reduced the prevalence of anaemia.

### 6.1. Strengths and Limitations of the Study

This study is multidimensional in nature as it captured demographic, biochemical, anthropometric, dietary, and parasitological data of the participants. The data collected established the household characteristics, nutritional and infection status, and dietary intakes of the participants at baseline. This is a pilot study limited to children aged 4–9 years who were participating in the Ghana School Feeding Program. For this reason, the findings cannot be extended to children outside this age range. The findings cannot be generalized to out-of-school children and pupils who do not participate in the Ghana School Feeding Program. Another limitation of the study is the inability to measure markers of inflammation such as C-reactive protein to correct for the influence of inflammation or infections on serum retinol concentrations of participants.

## Figures and Tables

**Table 1:** Background characteristics of participants by the study group.

Characteristics	Intervention (*n* = 53)	Control (*n* = 51)	*p* value
Mean age (years)	7.3 ± 1.7	6.7 ± 1.8	0.081

Sex			
Males	41.5	51.9	0.242
Females	58.5	47.1	0.176

Anthropometry			
Weight (Kg)	23.0 ± 4.9	21.6 ± 3.9	0.088
Height (cm)	117.1 ± 11.3	120.6 ± 11.3	0.110
WAZ-score	−0.731 ± 0.906	−0.485 ± .989	0.189
HAZ-score	−0.723 ± 1.149	−0.592 ± 1.17	0.567
WHZ-score	−0.121 ± 1.362	−0.095 ± 1.41	0.398
BMI-for-age *z* score	−0.388 ± 0.899	−0.105 ± 0.908	0.113

Malnutrition			
All category	34.0	41.2	0.051
Stunting (%)	13.2	17.6	0.530
Wasting (%)	11.3	15.7	0.084
Thinness (%)	5.7	3.9	0.381
Overweight (%)	3.8	2.0	0.681
Obesity (%)	0.0	2.0	0.068

Anaemia			
All category	41.5	37.3	0.055
Mild	32.1	33.3	0.998
Moderate	9.4	3.9	0.051

Low vitamin A level			
All category	66.0	64.7	0.087
Mildly low	26.4	27.5	0.995
Moderately low	24.5	17.7	0.053
Severely low	15.1	19.6	0.087

Infection status			
Malaria parasitaemia (%)	34.0	37.3	0.726

Guardian's age (years)			
20–60	100.0	98.0	0.474
≥61	0.0	2.0	0.197

Daily food consumption pattern			
Twice	1.9	0.0	0.199
≥thrice	98.1	100.0	0.344

Parental monthly income $ (GH¢)			
≤$178 (GHȻ499)	96.2	92.2	0.371
≥$178 (GHȻ500)	3.8	7.8	0.237

Food taboos			
Yes	72.5	64.2	0.362
No	27.5	35.8	0.334

Parental education			
Formal education	96.2	94.1	0.712
No formal education	3.8	5.9	0.891

Household backyard garden			
Yes	22	15.7	0.371
No	78	84.3	0.398

Dietary diversity score			
Low (≤3 food groups)	15.0	13.7	
Medium (4–5 food groups)	71.7	76.5	
High (≥6 food groups)	13.2	9.8	

*p* values obtained by the independent *t*-test, otherwise chi-square test, are significant at *p* < 0.05.

**Table 2:** Factors associated with anaemia in the study participants at baseline.

Factor	Odds ratio	95% CI	*p* value
Parental education status			
At least secondary education	Reference [1]		
Basic education	2.37	0.27–20.52	0.433
No education	1.57	0.47–5.28	0.468

Parental occupation			
Formal employment	Reference [1]		
Informal employment	1.027	0.360–2.932	0.960

Parental marital status			
Single	Reference [1]		
Married	2.50	0.16–3.02	0.511

Parental monthly income			
Monthly income (≥1000 cedis)	Reference [1]		
Monthly income (500–999 cedis)	1.79	0.019–2.40	0.210
Monthly income (1–499 cedis)	1.95	0.221–0.86	0.039

Participant's nutritional status			
Normal retinol level (≥20 *μ*g/dl)	Reference [1]		
Low retinol level (<20 *μ*g/dl)	1.68	0.10–0.99	0.049
Normal (HAZ > −2 standard deviations	Reference [1]		
Stunted (HAZ ≤ −2 standard deviations	1.59	0.13–2.64	0.491

Participant's infection status			
Malaria parasitaemia absent	Reference [1]		
Malaria parasitaemia present	1.64	0.27–9.89	0.069

Dietary intake			
Adequate intake (≥DRI)	Reference [1]		
Inadequate iron intake (<DRI)	2.32	0.48–3.63	0.052
Inadequate fat intake (<DRI)	1.03	0.99–1.07	0.127
Inadequate protein intake (<DRI)	1.23	0.13–8.67	0.431

Gender			
Boys	Reference [1]		
Girls	2.58	0.94–7.08	0.920

Age	0.82		0.738

*p* values are significant at *p* < 0.05. Binary logistic analysis was performed (Cox & Snell *R* Square = 0.186; Nagelkerke *R* Square = 0.248).

**Table 3:** Composition of the stews and soups consumed by study participants.

Nutrient composition of stews and soups/100 g as consumed								
Stew/soup	Group	Moisture (%)	Ash (mg)	Protein (g)	Fat (g)	Iron (mg)	Zinc (mg)	*β*-Carotene (mg)
Tomato stew	Control	70.7 ± 0.2	30.1 ± 1.5	4.6 ± 0.5	9.9 ± 0.5	3.0 ± 0.2	2.4 ± 0.5	2.8 ± 0.6
Tomato + MGLVP stew	Intervention	68.5 ± 0.4	32.3 ± 2.4	7.7 ± 0.1	10.1 ± 0.7	9.7 ± 0.1	7.8 ± 0.1	6.2 ± 0.5
Bean stew	Control	63.7 ± 1	15.5 ± 1.3	8.5 ± 1.7	10.0 ± 1.1	8.15 ± 1.1	4.3 ± 0.1	1.1 ± 0.3
Bean + MGLVP stew	Intervention	62.3 ± 2	17.2 ± 1.4	15.9 ± 1.4	12.1 ± 0.9	14.5 ± 1.3	8.1 ± 0.7	5.9 ± 0.7
Ground nut soup	Control	77.5 ± 1.2	21.3 ± 2.7	5.7 ± 0.2	15.1 ± 1.0	2.8 ± 0.2	1.1 ± 0.2	1.7 ± 0.3
Ground nut + MGVLP soup	Intervention	72.2 ± 1.0	25.2 ± 1.9	10.9 ± 0.6	16.4 ± 0.9	6.9 ± 0.1	5.8 ± 0.2	5.1 ± 1.1

*p* values (except that for fat) obtained by one-way ANOVA are significant between control and corresponding intervention meals at *p* < 0.05. Data presented are from laboratory analysis.

**Table 4:** Nutrient intake of study participants in comparison with Dietary Reference Intakes (DRIs) at baseline and end of the study.

Nutrient	Study group				*p* value
	Intervention		Control		
	Intake (mean ± SD)	Met DRI	Intake (mean ± SD)	Met DRI	
Protein (g)					
Baseline	32.7 ± 8.6	36 (67.9)	32.2 ± 9.5	33 (64.7)	0.793
End line	38.1 ± 9.1	45 (84.9)	32.6 ± 9.2	31 (66.0)	0.004^*∗*^
*p* value	0.001^*∗*^	0.003^*∗*^	0.954	0.875	

Iron (mg)					
Baseline	14.2 ± 7.1	32 (60.4)	13.7 ± 6.1	32 (62.7)	0.680
End line	24.1 ± 10.9	48 (90.6)	14.8 ± 6.2	39 (83.0)	0.001^*∗*^
*p* value	0.001^*∗*^	0.004^*∗*^	0.433	0.006^*∗*^	

Beta-carotene (*μ*g)					
Baseline	215 ± 23	23 (43.4)	211 ± 20	21 (41.2)	0.065
End line	694 ± 33	50 (94.3)	418 ± 35	28 (60.9)	0.001^*∗*^
*p* value	0.001^*∗*^	0.001^*∗*^	0.001^*∗*^	0.011^*∗*^	

Zinc (mg)					
Baseline	5.7 ± 2.1	22 (41.5)	5.4 ± 2.1	26 (51.0)	0.538
End line	13.8 ± 8.2	45 (89.4)	5.9 ± 2.3	25 (53.2)	0.001^*∗*^
*p* value	0.001^*∗*^	0.03^*∗*^	0.185	0.231	

Vitamin C (mg)					
Baseline	23.8 ± 3.8	11 (20.8)	23.4 ± 4.7	10 (19.6)	0.673
End line	24.2 ± 3.9	12 (22.6)	24.±4.7	9 (19.1)	0.287
*p* value	0.138	0.205	0.778	0.865	

Folic acid (mg)					
Baseline	30.5 ± 17.0	0 (0)	30.4 ± 12.2	0 (0)	0.971
End line	30.7 ± 16.9	0 (0)	30.8 ± 12.3	0 (0)	0.816
*p* value	0.844	—	0.839	—	

^*∗*^ *p* values within (across rows) and between (in most right column) study groups are significant at *p* < 0.05 by the paired and independent *t*-tests, respectively, otherwise by the chi-square test. The number of study participants at baseline was intervention = 53 and control = 51 and at end line was intervention = 53 and control = 47. Met DRI is the number (*n*) and percentage (%) of participants that met the various Recommended Nutrient Intakes. Nutrient intake was assessed by combination of 24-hour recall and direct food weighing (foods served at lunch break) (DRI source: National Research Council 1989, Recommended Dietary Allowances: 10th edition, Washington, DC: The National Academies Press (https://doi.org/10.17226/1349)).

**Table 5:** Nutritional and infection status of study participants at the end of the study.

Outcome variable	Intervention (*n* = 53)	Control (*n* = 47)	*p* value
Malnutrition			
All category	32.1	38.3	0.051
Stunting (%)	11.3	14.9	0.063
Wasting (%)	9.4	14.9	0.052
Thinness (%)	5.7	4.2	0.567
Overweight (%)	5.7	2.1	0.074
Obesity (%)	0.0	2.1	0.067

Anaemia			
All category (%)	28.3	53.3	0.024
Mild (%)	24.5	46.8	0.022
Moderate (%)	3.8	4.3	0.791

Low vitamin A			
All category (%)	20.8	23.4	0.059
Mildly low	20.8	17.0	0.055
Moderately low (%)	0.0	4.3	0.051
Severely low (%)	0.0	2.1	0.073

Infection status			
Malaria parasitaemia	39.6	40.4	0.935

Using Pearson's chi-square test for categorical variables, statistical significance is set at *p* < 0.05. The number of study participants at end line was intervention group = 53 and control group = 47.

**Table 6:** Food groups consumed by ≥50% of participants by the dietary diversity tertile at baseline and during the intervention study.

Tertile	Food groups
Lowest dietary diversity (≤3 food groups)	Cereals^*∗*^, meat^+^, poultry^+^ and fish^*∗*^, vegetables
Medium dietary diversity (4–5 food groups)	Cereals, meat^+^, poultry^+^ and fish^*∗*^, vegetables^*∗*^ and fruits^+^, roots and tubers, legumes^*∗*^, nuts and seeds
High dietary diversity (≥6 food groups)	Cereals, roots and tubers, meat^+^, poultry^+^ and fish^*∗*^, vegetables^*∗*^ and fruits^+^, fats^+^ and oils^+^, legumes^*∗*^, nuts and seeds

^*∗*^Most consumed food items in a food group. ^+^Most scarcely consumed food items in a food group.

## Data Availability

All data available have been included in this manuscript.

## Abbreviations

ACSMLVF: *Amaranthus cruentus* and *Solanum macrocarpon* leafy vegetable flour
RDA: Recommended Dietary Allowance
GSFP: Ghana School Feeding Program
RDI: Recommended Daily Intake
IRB: Institutional review board
GHȻ: Ghana cedis.
